# Tractography of the Brainstem in Major Depressive Disorder Using Diffusion Tensor Imaging

**DOI:** 10.1371/journal.pone.0084825

**Published:** 2014-01-21

**Authors:** Yun Ju C. Song, Mayuresh S. Korgaonkar, Lucy V. Armstrong, Sarah Eagles, Leanne M. Williams, Stuart M. Grieve

**Affiliations:** 1 The Brain Dynamics Center, Sydney Medical School, The University of Sydney and Westmead Millennium Institute, Sydney, NSW, Australia; 2 Discipline of Psychiatry, Sydney Medical School, The University of Sydney, Westmead Hospital, Sydney, NSW, Australia; 3 Department of Psychiatry and Behavioral Sciences, Stanford University, Stanford, California, United States of America; University of Illinois at Chicago, United States of America

## Abstract

**Background:**

The brainstem is the main region that innervates neurotransmitter release to the Hypothalamic-Pituitary Adrenal (HPA) axis and fronto-limbic circuits, two key brain circuits found to be dysfunctional in Major Depressive Disorder (MDD). However, the brainstem’s role in MDD has only been evaluated in limited reports. Using Diffusion Tensor Imaging (DTI), we investigated whether major brainstem white matter tracts that relate to these two circuits differ in MDD patients compared to healthy controls.

**Methods:**

MDD patients (n = 95) and age- and gender-matched controls (n = 34) were assessed using probabilistic tractography of DTI to delineate three distinct brainstem tracts: the nigrostriatal tract (connecting brainstem to striatum), solitary tract (connecting brainstem to amygdala) and corticospinal tract (connecting brainstem to precentral cortex). Fractional anisotropy (FA) was used to measure the white matter integrity of these tracts, and measures were compared between MDD and control participants.

**Results:**

MDD participants were characterized by a significant and specific decrease in white matter integrity of the right solitary tract (p<0.009 using independent t-test), which is a “bottom up” afferent pathway that connects the brainstem to the amygdala. This decrease was not related to symptom severity.

**Conclusions:**

The results provide new evidence to suggest that structural connectivity between the brainstem and the amygdala is altered in MDD. These results are interesting in light of predominant theories regarding amygdala-mediated emotional reactivity observed in functional imaging studies of MDD. The characterization of altered white matter integrity in the solitary tract in MDD supports the possibility of dysfunctional brainstem-amygdala connectivity impacting vulnerable circuits in MDD.

## Introduction

Major depressive disorder (MDD) is the fourth most disabling medical condition worldwide (based on disability-adjusted life years) and is expected to be ranked second by year 2020 [1]. One of the most prominent neurobiological findings in MDD is the dysregulation of the Hypothalamic-Pituitary Adrenal (HPA) axis [2]. The HPA axis is a highly regulated system in which secretory cells in the paraventricular nucleus receive neuronal inputs from many regions, including the amygdala, hippocampus and brainstem [3]. Locus coeruleus neurons in the brainstem provide stimulatory noradrenergic input to the paraventricular nucleus, and this leads to the activation of HPA axis and cortisol secretion [4], [5]. The brainstem is the site of primary neurotransmitter innervation to both the fronto-limbic system and the HPA, and is hence a site of primary interest in understanding the pathophysiology of MDD. In this study, we applied Diffusion Tensor Imaging (DTI) tractography to map the specific brainstem pathways that connect these two systems and to evaluate structural changes with MDD.

Since the brainstem is a source for many of the neurotransmitters implicated in theories of MDD (including the HPA axis), brainstem structural alterations clearly have a potential mechanism by which they can play a role in the development of MDD. However, there is little evidence to support any structural basis for the involvement of this region in depression. Some limited reports describe volumetric brainstem changes in MDD cohorts [6]–[9]. A limited number of reports have evaluated brainstem white matter changes in MDD. These studies showed elevated fractional anisotropy (FA) in the substantia nigra of the brainstem [10]; greater mean diffusivity in the pons of the brainstem [11]; increased regional FA between the amygdala and the hippocampus, cerebellum and brainstem [12]; or failed to detect any significant abnormalities [13]. These studies were somewhat limited by small sample size and by the imprecise anatomical localization provided by the methods used, which do not target specific tracts.

Recent work using DTI, including data from our group, has shown that altered structural white matter connectivity in the fronto-limbic systems distinguishes MDD from healthy controls [14]. DTI tractography is an advanced Magnetic Resonance Imaging (MRI) technique that permits quantification of these circuits. It involves first ‘seeding’ a region of interest, then identifying the specific white matter fibres that connect to a target region. In this study, we used probabilistic tractography to characterise the white matter tracts that connect the brainstem to the striatum, amygdala and frontal circuitry in a large, well-characterised cohort of participants with MDD and controls. Our cohort was drawn from the imaging sub-study of the International Study to Predict Optimized Treatment for Depression (iSPOT-D) [15]. We specifically targeted three white matter brainstem fiber tracts which we hypothesized to have a key involvement in the pathophysiology of MDD. The nigrostriatal tract is one of the major dopamine tracts that link the brainstem to the striatum. The corticospinal tract is a major white matter tract running from the spinal cord through the brainstem to the precentral cortex. The solitary tract projects from the medulla to the amygdala. These tracts all project from the brainstem to regions that are strongly implicated and associated with the HPA axis in MDD also encompassing the dopaminergic, serotenergic and noradrenergic systems known to be vulnerable in MDD.

We predicted that the FA for these selected brainstem tracts would differ between MDD participants and controls.

## Materials and Methods

### Ethics statement

This study was conducted according to the principles of the Declaration of Helsinki 2008 and approved by the institutional review board (Western Sydney Local Health District Human Research Ethics Committee). After study procedures were fully explained in accordance with the ethical guidelines of the institutional review board, participants provided written informed consent.

### iSPOT-D enrollment and protocol

MDD and control participants aged 18–65 years were recruited from the general population in the greater Sydney area. All MDD participants were diagnosed for major depression by their primary care providers and were confirmed to meet the DSM-IV criteria for single or recurrent nonpsychotic MDD established by MINI Plus, and a 17-item Hamilton Rating Scale for Depression (HRSD_17_) score ≥16. They did not have suicidal tendencies, history of bipolar, schizophrenia, schizoaffective disorder or psychosis, current primary diagnosis of anorexia, obsessive-compulsive disorder or primary post traumatic disorder. All participants also did not have substance dependence, history of brain injury or suffer loss of consciousness for greater than five minutes or any contraindications for MRI. A complete description of the iSPOT-D study protocol, clinical assessments, inclusion/exclusion criteria and diagnosis procedures of the overall trial is provided in Williams et al. [15].

All MDD participants were anti-depressant treatment naïve or had undergone an anti-depressant wash-out period. Participant age of onset and disease duration (years) were documented for analyses.

### Participants in the present study

DTI data were drawn from the first 102 MDD participants and 34 age- and gender-matched healthy controls (18–65 years of age) who provided MRI data at the primary imaging site—Westmead Hospital, Sydney, Australia—as part of iSPOT-D baseline data collection. These 102 MDD participants represent the first 50% of participants entering the study and locked for analysis as per the analysis plan for the study (the analysis plan for the study has been described in detail elsewhere [15], [16]. DTI data for seven MDD participants were not collected at baseline due to time constraints, resulting in 95 MDD participants for analysis.

### Image acquisition

Magnetic resonance images were acquired using a 3.0 Tesla GE Signa HDx scanner (GE Healthcare, Milwaukee, Wisconsin). Acquisition was performed using an 8-channel head coil. Diffusion tensor images were acquired using a spin-echo DTI-Echo Planar Imaging sequence. Seventy contiguous (no gap) 2.5 mm slices were acquired in an axial orientation with an in-plane resolution of 1.72 mm×1.72 mm and a 128×128 matrix (TR: 17000 ms; TE: 95 ms; Fat Saturation: ON; NEX: 1; Frequency direction: R/L). A baseline image (b = 0) and 42 different diffusion orientations were acquired with a b-value of 1250 s/mm^2^. Total acquisition time for the DTI protocol was 13 min 36 s. In addition to the DTI, participants also performed a T1 weighted scan, a PD/T2 weighted scan, and five functional MRI scans at baseline [16]. However data from these other scans were not used in the current study.

### DTI data analysis

**Figure 1:**
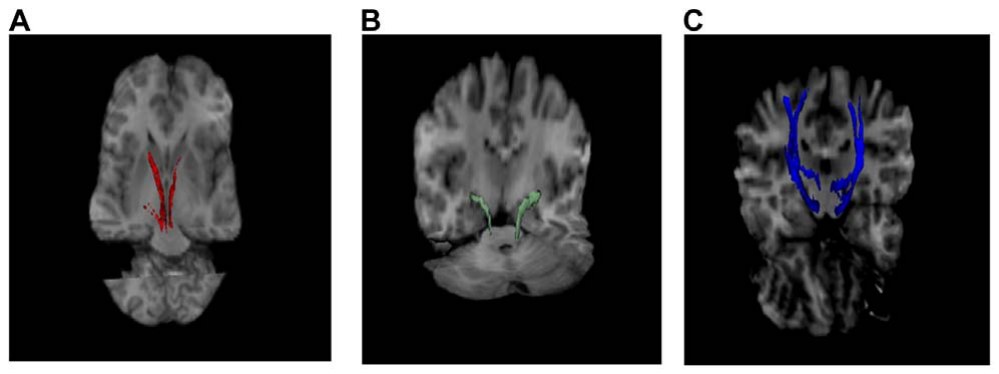
The three brainstem tracts identified using probabilistic tractography. A: Nigrostriatal tract (Left and Right in red). B: Solitary tract (Left and Right in green). C: Corticospinal tract (Left and Right in blue) superimposed over a representative DTI image.

The DTI data processing and analytic methods have been described in detail elsewhere [14], [17]. DTI data was preprocessed and analyzed using the Oxford Centre for Functional MRI of the Brain (FMRIB) Diffusion Toolbox from the FMRIB Software Library release 4.1.3 (http://www.fmrib.ox.ac.uk/fsl). Briefly, the raw DTI data for each participant were first corrected for head movement and eddy current distortions. A binary brain mask was generated using the baseline non-diffusion weighted (b = 0) image. Diffusion tensor models were then fitted independently for each voxel within the brain mask and fractional anisotropy was calculated for all brain voxels. Next, probabilistic tractography was performed in native space for each participant. Probabilistic tractography was used to parcellate the tracts of interest, a method that has been described elsewhere [14]. Briefly, this method is based on probability distributions that guide multiple fiber samples starting from a seed voxel to a specified target region. Also, this method models multiple fiber orientations passing through the same voxel and has been shown to be robust to resolving crossing fibers which is critical for tractography in the brainstem [18]. Tractography was performed using the brainstem mask as the seed and the striatum (nigrostriatal tract), amygdala (solitary tract) and precentral cortex (corticospinal tract) as the target regions. For the brainstem, a single axial slice approximately at the end of the midbrain and before the pons was used to outline the brainstem and used as seed mask for each individual participant (refer to Supplementary Figure S1). For the target regions, the regions were identified using the freesurfer analysis of the participant’s structural T1 weighted MRI data and were used as masks (described in detail in [14]). One thousand fiber streamlines were generated from each voxel within the seed region, and only those that reached the target region were retained as the final white matter connection. The streamlines were terminated once they reached a target region. Only those voxels with at least 40% of the maximum streamlines passing through them were retained as part of the final white matter tract. Tractography for the three tracts of interest (left and right separately) were performed and quality checked visually by three authors for all participants (Figure 1). The mean FA value for each tract was then calculated.

### Statistical Analyses

An independent samples t-test was used to compare mean differences in FA values between the MDD and control groups. For the MDD group, Pearson’s correlations were calculated between FA measures and age of disease onset, disease duration and baseline HRSD_17_ severity. To control for any type 1 errors due to multiple comparisons, a modified threshold of p<0.016 (0.05/3 for three tracts of interest) was used.

## Results

### Participant Characteristics

Clinical and demographic features are presented in Table 1. The MDD participant and control groups did not differ significantly in age or gender. As expected, the groups did differ significantly in their baseline HRSD_17_ severity (p<0.001).

**Table 1:** Demographic characteristics.

Characteristic	Controls (n = 34)	MDD (n = 102)
Age (years)	31.5±12.4	33.8±13.1
Gender	18M∶16F	48M∶54F
HRSD_17_ Baseline*	1.0±1.2	21.0±3.9
Age of Onset (years)	-	22.1±12.2
Disease duration (years)	-	11.3±11.8

* Difference between MDD & Control at p<0.001.

Abbreviations: HRSD_17_, 17-item Hamilton Rating Scale for Depression; MDD, Major Depressive Disorder.

### FA differences between MDD participants and controls

The independent samples t-test revealed significant group differences in mean FA values for the right solitary tract in MDD when compared to controls (2.662 [118], p = 0.009, Table 2). No significant differences were present for the other tracts. There was no significant correlation between tract FA and age of disease onset, disease duration or baseline HRSD_17_ severity.

**Table 2:** Tractography values.

Tracts	Controls	MDD	t (df)	p-value
	(Mean ± SD)	(Mean ± SD)		
Nigrostriatal Left				
FA	.5118±.0379	.5139±.0347	−0.273 (118)	0.785
AD	.0013±.0001	.0013±.0001	0.144 (118)	0.885
RD	.0005±.0000	.0005±.0000	0.341 (118)	0.734
MD	.0008±.0000	.0008±.0000	0.357 (118)	0.722
Nigrostriatal Right				
FA	.5023±.0340	.5101±.0331	−1.101 (118)	0.273
AD	.0013±.0001	.0013±.0001	−0.648 (118)	0.518
RD	.0005±.0000	.0005±.0001	0.576 (118)	0.566
MD	.0008±.0000	.0008±.0000	−0.045 (118)	0.964
Solitary Left				
FA	.4475±.0505	.4327±.0588	1.233 (118)	0.220
AD	.0014±.0001	.0014±.0001	−0.642 (118)	0.522
RD	.0007±.0001	.0007±.0001	−1.620 (118)	0.108
MD	.0009±.0001	.0009±.0001	−1.381 (118)	0.170
Solitary Right				
FA	.4861±.0552	.4545±.0567	2.662 (118)	0.009*
AD	.0014±.0001	.0014±.0001	0.705 (118)	0.482
RD	.0006±.0001	.0007±.0001	−1.866 (118)	0.065
MD	.0009±.0001	.0009±.0001	−1.070 (118)	0.287
Corticospinal Left				
FA	.5651±.0343	.5663±.0366	−0.148 (118)	0.883
AD	.0013±.0001	.0013±.0001	.370 (118)	0.712
RD	.0006±.0000	.0006±.0000	-0.039 (118)	0.969
MD	.0008±.0000	.0008±.0000	0.085 (118)	0.932
Corticospinal Right				
FA	.5704±.0647	.5859±.0336	−1.681 (118)	0.096
AD	.0013±.0001	.0013±.0001	−0.206 (118)	0.837
RD	.0005±.0001	.0005±.0000	1.066 (118)	0.288
MD	.0007±.0000	.0007±.0000	0.849 (118)	0.397

* significant p–value.

Abbreviations: FA, Fractional Anisotropy; AD, Axial Diffusivity; RD, Radial Diffusivity; MD, Mean Diffusivity MDD, Major Depressive Disorder.

## Discussion

In this study, we used probabilistic tractography to characterize three white matter tracts from the brainstem (nigrostriatal tract, solitary tract and corticospinal tract) in a well-defined population of participants with MDD and controls. Our data showed decreased connectivity in MDD that was specific to the right solitary tract, where the FA was decreased by 9.3% compared to control participants (p<0.01). This is the first demonstration of a specific tract-based abnormality in the brainstem in MDD, providing a structural basis for “bottom-up” involvement of the brainstem in this condition. Our study has important implications for understanding the mechanisms of MDD, and for the design of measures that may permit better diagnosis and treatment prediction.

Of the three tracts we hypothesized to have an involvement in MDD, only the right solitary tract was found to have significantly reduced FA in MDD compared to controls. This result is compelling as the solitary tract connects the brainstem to the amygdala, a region commonly implicated in MDD and critical in regulating the HPA axis. The HPA axis is an important circuit in understanding stress response, particularly in MDD where the hippocampus, amygdala and prefrontal cortex are all implicated; all of these regions are considered to play a role in regulating this circuit [19]. The amygdala plays a role in stimulating corticotrophin-releasing hormone (CRH) and cortisol secretion during stress [20]. Dopaminergic, noradrenergic and serotonergic terminals are distributed in these amygdala CRH neurons, with the noradrenergic terminals originating from the nucleus of the solitary tract [21]. Cell bodies of the noradrenergic neurons in the brainstem that project to the basolateral amygdala contain one of the highest densities of glucocorticoid receptors known in the mammalian brain [22], cell systems that are vital in regulating the HPA axis.

The link between HPA dysfunction and MDD is supported by both human and animal data. Post-mortem studies have shown abnormalities in glucocorticoid receptors in patients with severe mood disorders [3]. A recent mouse model has also shown that a disruption of glucocorticoid receptors mimics hyperactivity of the HPA axis, impaired negative feedback regulation of the HPA axis and depression-like behavior [23]. These studies provide an interesting context to our findings, suggesting the potential importance of the connection between the brainstem and amygdala via the solitary tract in MDD.

Chronic stress has been associated with the development of MDD [24]. Joint activation of the amygdala and brainstem structures during rest has been described to constitute an intrinsic connectivity network that participates in continuous salience processing within the homeostatic, emotional and cognitive domain [25]. Observations of enhanced resting state connectivity between the amygdala and brainstem under mild psychological stress conditions support the functional importance of the amygdala-brainstem circuit in the response to stressful stimuli [26]. The connectivity between the brainstem and the amygdala may therefore be critical to the maintenance of an appropriate stress response. Decreased structural connectivity in this circuit in MDD, as found in our results, could potentially impair the normal stress response and thereby contribute to the development and maintenance of a depressed state. To note that we found decreased structural connectivity whereas enhanced intrinsic functional connectivity between the brainstem and amygdala has been reported. Further work is needed to understand this structure-function link in connectivity between regions. Functional MRI data has also been collected on the same cohort of participants and will allow us to evaluate these links in more detail in future work.

The lateralization of reduced FA in the right solitary tract is an intriguing result. The exact significance of this finding is requires further evaluation however it is consistent with some of the other findings of lateralization in depression. Functional imaging studies also demonstrate laterality in depression, where the left amygdala to be is hyporeactive to negative non-conscious emotion processing [16], whereas the right tends to have greater activity during conscious emotion processing [27]. In the context of this data, and given the anatomical link between the solitary tract and the amygdala, our result provides a new insight into the role of the white matter pathways associated with the amygdala in depression.

The involvement of the brainstem in MDD has been largely overlooked by previous structural imaging studies, despite the known disruption of neurotransmitter systems in MDD with anti-depressant treatments designed to stabilize neurotransmitter instability [28]. While the relevance of the solitary tract to MDD is poorly understood, the literature contains several clues that provide some support for a key role in treatment. Vagal nerve stimulation is a well-established effective therapy for treatment-resistant depression that targets the solitary tract [29], [30] with the aim of altering persistent autonomic and somatic symptoms of depression. This mechanism of this therapy is considered to be primarily via action on the locus coeruleus and its modulation of cerebral blood flow in the amygdala, dorsolateral prefrontal cortex and cingulate cortex [31]–[33]. Stimulation of the solitary tract evokes currents in the cardiac vagal neurons, thus having direct effects on heart rate [34]. Reduced heart rate variability has been demonstrated in MDD, and has been targeted as a potential therapeutic target via biofeedback training [13], [35]. We anticipate that the combination of investigating imaging brainstem biomarkers and other physiological autonomic measures (e.g., Electro Dermal Activity, Heart Rate Variability) may further validate the importance of the solitary tract in the context of the role of amygdala-regulated stress circuits in treatment outcome.

The use of tractography to quantify FA values for the target tracts in the brainstem is a novel application in MDD that enables us to more specifically target putative tracts, permitting a hypothesis-driven analysis of the data. Our previous work has characterized white matter tracts in MDD utilizing the tract-based spatial statistical analysis and tractography of DTI [14], [17]. Using these whole-brain methods, we have been able to map inter-regional connectivity of the entire cortex, characterizing the abnormalities of circuits involved in MDD. This work has supported the theory of fronto-limbic dysfunction in MDD. Our present study extends this work by utilizing tractography to characterize the involvement of specific tracts that would not be anatomically resolved using a global method such as tract-based spatial statistical analysis. A potential limitation with using mean FA for the whole tract is the dilution of signal from the more subtle structural abnormalities, especially for longer tracts where these differences maybe more localized. However, this measure should be good representation of the overall white matter integrity in the connection between the two regions and has been used for evaluation of inter-regional connectivity across the whole brain [36].

A potential limitation of our study is inherent to DTI tractography in the brainstem and derives from EPI-related geometric distortion and local sensitivity loss due to signal drop out. While these factors are unlikely to influence our between cohort comparisons, it could impact the precision of tractography and therefore the FA estimate from each tract. The limited angular resolution of DTI limits its ability to track white matter in areas with crossing overlapping fibers. However, our use of probabilistic tractography partially compensates for this limitation due to the ability of this method to resolve crossing fibers [18]. Future studies using methods such as High Angular Resolution Diffusion-weighted Imaging or Diffusion Spectrum Imaging would improve angular resolution, and so greatly improve the evaluation of brainstem circuitry. We are currently extending our cortex-cortex connectivity analysis to include brainstem nuclei and other sub-cortical structures, an approach that is likely to provide particular insights into brainstem-cortex pathways that may impact fronto-limbic circuits in MDD [3].

In summary, our results highlight the importance of brainstem circuits in MDD. The identification of reduced white matter integrity in the right solitary tract in MDD provides solid support to the theory that altered brainstem function impacts on MDD via the HPA axis, with the amygdala as a key region modulating this effect. The present study places the brainstem back in the spotlight as a potential key region in modulating the development and maintenance of depression.

## Supporting Information

Figure S1 **Axial view of the brainstem mask (in red) in panel A.** Saggital view of the brainstem mask (in red) in panel B.
(TIF)
