# Supplementary figures and images for: Tractography of the Brainstem in Major Depressive Disorder Using Diffusion Tensor Imaging
Source: PLoS One. 2014 Jan 21;9(1):e84825. doi: 10.1371/journal.pone.0084825 (PMC3897382; doi:10.1371/journal.pone.0084825)

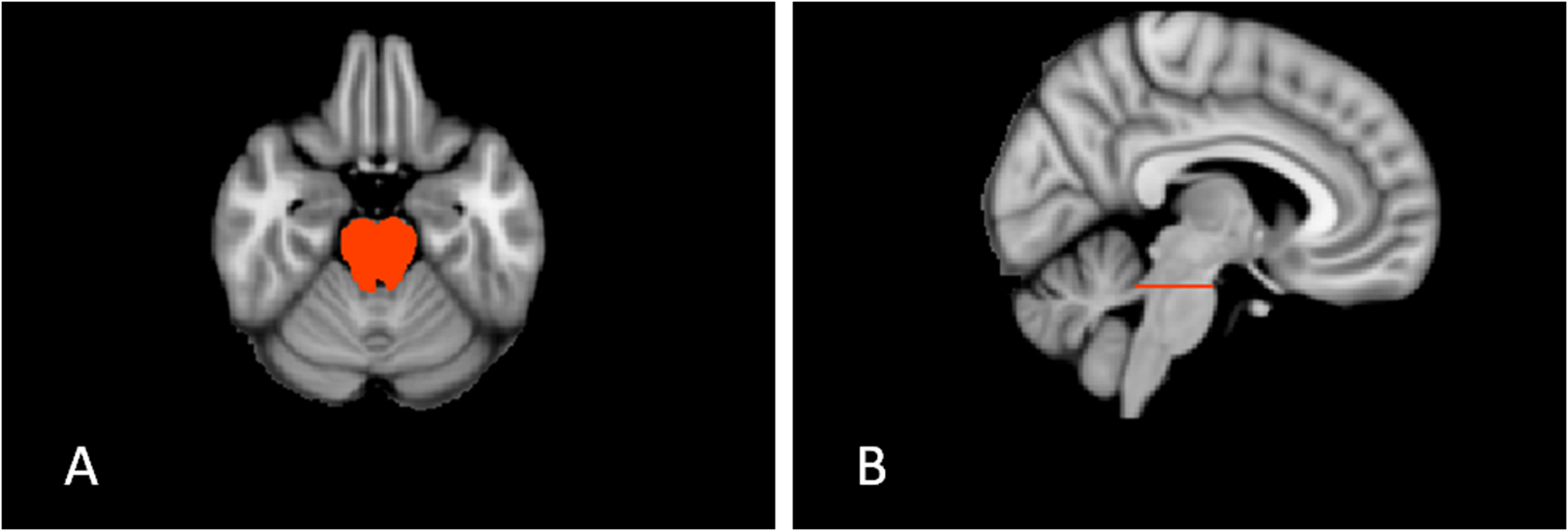

Supplement: Figure S1 — Axial view of the brainstem mask (in red) in panel A. Saggital view of the brainstem mask (in red) in panel B. (TIF) [file pone.0084825.s001.tif]
